# When Load is Low, Working Memory is Shielded From Long-Term Memory’s Influence

**DOI:** 10.5334/joc.368

**Published:** 2024-05-15

**Authors:** Lea M. Bartsch, Gidon T. Frischkorn, Peter Shepherdson

**Affiliations:** 1 Univeristy of Zurich, CH; 2 Univeristy of Akureyri, IS

**Keywords:** Working memory, Long-term memory, Proactive facilitation, Proactive interference

## Abstract

Previous studies found that episodic long-term memory (eLTM) enhances working memory (WM) performance when both novel and previously learnt word pairs must be retained on a short-term basis. However, there is uncertainty regarding how and when WM draws on eLTM. Three possibilities are (a) that people draw on eLTM only if WM capacity is exceeded; (b) that there is *always* a contribution of eLTM to WM performance, irrespective of whether prior knowledge is helpful or not; or (c) benefits of prior knowledge are specific to comparisons between conditions which are similarly ambiguous concerning whether LTM may be useful. We built on the assumption that under conditions of a contribution from LTM, these LTM traces of memoranda could benefit or hamper performance in WM tasks depending on the match between the traces stored in LTM and the ones to-be stored in WM in the current trial, yielding proactive facilitation (PF) and proactive interference (PI), respectively. Across four experiments, we familiarized participants with some items before they completed a separate WM task. In accordance with possibility (a) we show that there are indeed conditions in which only WM contributes to performance. Performance deteriorated with the addition of stimuli from eLTM when WM load was low, but not when it was high; and an exchange of information between LTM and WM occurred only when WM capacity was exceeded, with PI and PF effects affecting immediate memory performance in verbal and visual tasks only at higher set sizes.

Working memory (WM) is the system responsible for holding the information that is currently most relevant available for processing. It has a limited capacity and enables quick access as well as the construction of new representations, allowing people to create new thoughts, form complete sentences, and plan future actions (Baddeley, 1986; Cowan, 2008). The system’s capacity limit has been identified as a key constraint on human cognition, and individual differences in WM capacity are associated with meaningful real-world outcomes in domains such as reading (Daneman & Carpenter, 1980), educational performance (Alloway & Alloway, 2010), and decision-making (Bechara & Martin, 2004). Specifically, evidence suggests that indicators of a person’s ability to maintain relations between items, or relations between items and their context (i.e., bindings) are highly correlated with fluid intelligence (Wilhelm et al., 2013), leading to the proposal of the binding hypothesis of WM (Oberauer, 2009), which states that WM capacity is limited by the number of bindings a person can form and maintain. Similarly, WM capacity has been estimated to reflect a limited number of (three or four) chunks which can be retained, (Chen & Cowan, 2009; Cowan, 2001). Such chunks can compress information that would otherwise (i.e., if not chunked) occupy a greater share of capacity – for instance the individual letters “P” “D” and “F” can be stored in WM as a single chunk consisting of “PDF”.

As becomes apparent by this example, WM is not assumed to be independent from information stored more permanently in our long-term memory (LTM). In fact, current models of WM assume a close interaction with prior knowledge. These models conceptualize WM as a subset of LTM representations each of which is – for a limited time – in a heightened state of accessibility (Cowan, 1995; McElree, 1998; Oberauer, 2002). In contrast to WM’s limited capacity, LTM has the advantage that its potentially unlimited content can be accessed indefinitely– if sufficiently distinct retrieval cues are available (Schacter & Squire, 1996; Surprenant & Neath, 2013; Tulving, 1983). Despite the theoretical connection of LTM and prior knowledge with WM, the conditions under which episodic LTM contributes to performance in WM tasks remain unclear.

## Contribution of prior knowledge from episodic LTM to WM

When investigating the contribution of LTM to WM, it is important to distinguish between semantic and episodic LTM (see Rubin, 2021). Episodic LTM refers to remembering the personal experience of past events. This can be the memory of dinner with friends last night, but can also include, for example, experiences individuals have been exposed to in an experiment, including memory for lists of words or pictures presented to them. Semantic LTM refers to our knowledge of facts, including the meaning of words, and representations of well-known concepts. Knowledge from semantic LTM is assumed to contribute to performance in most tests of WM, as shown in effects like the sentence superiority benefit and the lexicality effect (e.g. Baddeley et al., 2009). These effects from semantic LTM are usually explained through redintegration, the process of reconstructing a degraded memory trace (Hulme et al., 1997). There is consensus that semantic LTM contributes generally to performance in tests of WM (see e.g., Asp et al., 2021; Brady et al., 2016; Jackson & Raymond, 2008; Xie & Zhang, 2017).

However, the nature of contributions from *episodic* LTM are still unclear, including how far they differ from semantic contributions and *when* WM relies on episodic traces. Previous research indicates two ways in which episodic LTM could contribute to WM tasks: (1) through episodic traces which are rapidly created in the activated part of LTM and contribute to performance (Cowan, 2019) and (2) via pre-learnt representations that could influence the build-up of representations in the activated part of LTM (e.g. Chen & Cowan, 2005). The former corresponds to findings that episodic memories are formed within a few seconds (Huebner & Gegenfurtner, 2011; Verhaeghen et al., 1998). This means that the timing of a typical WM task is sufficient to lay down an episodic memory trace of the to-be-remembered information. Indeed, previous research has shown that performance in immediate memory tasks can be driven by both WM and episodic LTM (Bartsch & Oberauer, 2023; Raaijmakers & Shiffrin, 1980; Unsworth & Engle, 2006a). Yet this leaves us with a theoretical conundrum: Why, if episodic LTM can be used to support memory in a WM test, is performance in these tests subject to a capacity limit? – or in other words, why are subjects not using their episodic memory all the time? One factor that gives rise to this lies in the core function of WM: to hold current goals and information to guide behavior. These can deviate crucially from (very similar) episodic traces stored in our unlimited LTM (e.g., remembering a friend’s new phone number in order to call them back). Therefore, these representations need to be shielded against proactive interference from LTM. In conclusion, the contributions of prior knowledge to WM must be limited to specific conditions and the goal of the present work is to investigate when WM draws on episodic LTM to benefit performance.

As noted above, further evidence for a contribution of prior knowledge from episodic LTM to WM comes from literature showing superior performance by experts on tasks that make use of their knowledge (e.g., Ericsson & Kintsch, 1995). Chase and Simon (1973) showed that chess experts – having stored a large number of specific patterns of chess pieces in LTM – clearly outperformed novices in an immediate memory test for chess positions. The researchers hypothesized that experts’ extensive and well-practiced prior knowledge allowed them to rapidly recognize these, driving their superior performance. Consistent with their hypothesis, the experts’ advantage disappeared when chess boards of randomly arranged chess pieces were used as stimuli (Charness, 1991). Inspired by these findings, Ericsson and Kintsch (1995) proposed a theory called long-term working memory (LT-WM) that specifies how WM is transformed by prior knowledge. They hypothesized that experts learn to use episodic LTM as a substitute for normal WM, circumventing its usual limits. Within the theory, LT-WM was not considered an extra memory system, but a functionality that allowed experts to use episodic LTM more efficiently in situations requiring WM – such as the situation of planning the next couple of chess moves based on the possible future turns of an opponent.

Taken together, LTM should aid in bypassing WM’s capacity limit and there are two potentially connected ways in which LTM has been shown to benefit WM: (1) through better memory for the items matching LTM representations themselves (e.g., rapidly formed episodic traces or a pre-learnt pair of words; see Bartsch & Shepherdson, 2022, 2023; Chen & Cowan, 2005, 2009) and (2) by subsequently freeing up capacity for new stimuli (Mizrak & Oberauer, 2020; Norris et al., 2019; Thalmann et al., 2019).

## Working memory capacity and the contribution of LTM to WM performance

Measures of WM capacity have been shown to be highly correlated with the ability to remember over the long-term (Unsworth, 2010; Wilhelm et al., 2013) and other researchers have argued that responses in WM tasks result from a combination of representations maintained in the short-term buffer, and information retrieved from LTM (Raaijmakers & Shiffrin, 1980; Shiffrin & Atkinson, 1969; Unsworth & Engle, 2006a, 2006b, 2007a, 2007b). They argue that items in these tasks can be retrieved from LTM using temporal-contextual cues.

A key claim in this prior work is that the influence from episodic LTM becomes especially important in cases where the capacity of WM has been exceeded (McCabe, 2008; Unsworth & Engle, 2006a, 2006b, 2007a, 2007b). For instance, McCabe (2008) showed that in the presence of a distractor task the capacity of WM is exceeded, and performance of the WM task is influenced by episodic LTM. Other research has shown that when long lists need to be remembered in simple span tasks, individuals’ ability to draw on representations stored in LTM (secondary memory) becomes important. Specifically, Unsworth and Engle examined the relationship of performance in simple and complex span tasks to fluid abilities as a function of list-length, finding that only the longest simple span task shared a similar predictive relationship with fluid abilities to that of complex span tasks (Unsworth & Engle, 2006b). Taken together, this line of research suggests that information from LTM influences performance in WM tasks, and that this influence varies as a function of the task used and individual differences in WM.

To date, only a few studies have investigated how WM draws on available prior knowledge from episodic LTM, and under which conditions this functionality of WM occurs. For instance, Chen and Cowan (2005, 2009) showed that participants had better recall for lists consisting of pairs of words that had been learned in an earlier phase of the experiment, compared to lists with novel pairings of words. Critically, this benefit was more pronounced at larger set sizes (e.g., 12 to-be-remembered items) than at smaller ones (e.g., set size 8). This indicates that immediate recall of verbal lists benefits from LTM representations, and that WM load affects the magnitude of this benefit. The relevance of load is underlined by a recent study showing a double dissociation of contributions of WM and episodic LTM to performance in an immediate memory task for word-word pairs: At set sizes larger than 3, LTM strongly contributed to performance, whereas performance at set size 2 was driven by WM (Bartsch & Oberauer, 2023).

One plausible explanation for all of the above patterns is that people draw on episodic LTM if (and only if) WM capacity is exceeded (*WM overload hypothesis*). This would mean that the benefit of prior knowledge to WM happens only when some negotiation of information between WM and LTM is already necessary. A system of this sort, with some information able to be effectively quarantined from the unwanted influence of prior knowledge, would have major advantages for adaptive behavior – for instance, allowing individuals to process information that directly contradicts the contents of long-term memory where this is desirable. This hypothesis predicts that there are conditions of low memory load, in which performance is driven by representations stored in WM only, whereas at higher loads, subjects draw on both long-term and working memory. Thus, the effect of prior knowledge on WM performance would vary with the amount of new information that has to be maintained in WM overall. This is opposed to the view that there is *always* a contribution of LTM to WM performance (see Table 1 for the full hypothesis-space). This contribution could arise from episodic traces which are rapidly created in the activated part of LTM and contribute to performance (Cowan, 2019) or prior available knowledge, for which there would be no need for encoding a new representation into WM. The contribution of LTM to WM performance would hereby be independent of WM load.

**Table 1:** Hypotheses on the contribution of prior knowledge to WM organized by Experiment.


HYPOTHESIS	ALTERNATIVE HYPOTHESIS	EXPERIMENT

**Under which conditions does LTM contribute to performance in a WM task?**		

A) **Obligatory LTM hypothesis:** There is always a contribution of episodic LTM.	B) **WM overload hypothesis:** Once a person’s WM capacity is exceeded, performance is influenced by episodic LTM.	3 and 4

	C) **Decision ambiguity hypothesis:** Benefits of prior knowledge are specific to conditions in which there is no ambiguity about decisions of using WM or LTM.	2


One way to test predictions from these two hypotheses is to familiarize participants with a subset of the memoranda prior to a separate WM task – in which the amount of new information that needs to maintained is varied as well. The WM overload hypothesis would predict that if the amount of new information in the WM task is sub-WM-capacity, such that participants solely rely on WM to govern their responses, then performance should be affected by the addition of memoranda for which participants have prior knowledge: Adding these stimuli leads to a load which WM alone can no longer manage, requiring some level of reliance on LTM – which is less limited in capacity but more fallible with respect to proactive interference. Instead, if the amount of to-be-maintained new information exceeds WM capacity, such that people are already drawing on episodic traces rapidly built in the activated part of LTM (see Cowan, 2019), then WM performance should be unaffected by the addition of extra stimuli for which participants have prior knowledge.

The results of previous research are consistent with this prediction: Across a series of experiments participants completed a multi-alternative recognition test for small sets of word pairs (Bartsch & Shepherdson, 2022). The key manipulation involved the composition of the pairs contained in the sets. Sets consisted of either (a) some pairs that had been pre-learnt earlier in the session (LTM pairs), and some pairs that were new; or (b) new pairs only. Further, we varied the amount of information participants had to remember, either being sub-WM capacity (set size 2) or taxing WM capacity (set sizes 4, 6, or 8). In accordance with the above hypothesis that people draw on episodic LTM if (and only if) WM capacity is exceeded, the pattern of results in this study varied with the amount of new information that had to be maintained in WM: When WM load was low (WM load = 2 pairs), performance for the new pairs deteriorated with the addition of stimuli for which participants had prior knowledge (*2 + 2 condition*) but remained superior to performance with the matched set size comprising only new pairs. By contrast, when WM load was higher (WM load = 4 pairs), adding stimuli for which participants had prior knowledge did not deleteriously affect performance for the new pairs.

However, these results are also in line with the alternative explanation that there is always a contribution of LTM (*Obligatory LTM hypothesis*). For instance, in the condition including 2 LTM and 2 new pairs (*2 + 2 condition*) people may *sometimes* hold all 4 sets in WM, and at others rely on LTM as well. In general, one would get a cost of adding LTM pairs^1^ to the extent that these pairs are still encoded into WM, which happens less often the more WM is already full. Furthermore, new pairs could also be offloaded to LTM to free capacity for new information.

Lastly, this pattern of results could be explained by a second alternative hypothesis that benefits of LTM are specific to comparisons between conditions with equivalent ambiguity concerning whether stimuli need to be encoded into WM, or can be offloaded to LTM (*Decision ambiguity hypothesis, see* Table 1). Specifically, any benefits of prior knowledge in a given trial could be countered by the costs of having to determine whether a stimulus must be encoded into WM, or whether the relevant information already exists in LTM. When such decisions are necessary, those items with an existing LTM representation would either need to be prevented from reaching WM in the first place (i.e., filtered out) or removed from WM after encoding, both of which lead to costs for performance (e.g., Oberauer, 2018). In our previous work showing this pattern of results, set size 2 was the only list length for which participants knew they would have to rely exclusively on WM (and thus would not have to filter or remove any pairs). Critically, this was also the only condition where performance was superior relative to a condition with added LTM pairs (i.e., in the *2 + 2 condition*). By contrast, when four pairs were presented it was ambiguous whether a decision between WM and LTM representations had to be made (i.e., in the *2 + 2 condition*) or whether participants should rely exclusively on WM (i.e., in the *4 + 0 condition*), meaning that participants had to judge whether to filter or remove each pair as it was presented. For all other set sizes (6/4 + 2, and 8/4 + 4) in which memory for the new pairs did not deteriorate with the addition of LTM pairs, participants unambiguously knew they would have to decide about using WM or LTM representations. In sum, the comparison between the set size 2 condition, and the set size 4 condition consisting of 2 pre-learnt and 2 new pairs, was the only comparison between a condition where participants knew in advance that filtering/removal was *not* necessary, and one in which they knew it *might* be. Thus, the finding of a difference between those conditions, but not the others, may be explained by this inequality.

The goal of the present study is to directly test and adjudicate between these alternative hypotheses.

## The present study

In this series of experiments, we tested three different hypotheses pertaining to the conditions under which prior knowledge from LTM contributes to WM (see Table 1). In the first phase of each experiment, we presented participants with stimuli (word pairs Experiments 1, 2, and 3; color-object conjunctions Experiment 4) for them to encode into LTM (*LTM learning phase*). Subsequently, they completed trials of a WM task, also involving the same type of stimuli. The pairs/conjunctions presented in each WM trial consisted of varying numbers of new pairs (LTM unavailable) and the previously learned LTM pairs. Crucially, WM load was varied to be low or high.

In the first experiment we examined whether the pattern of results of the previous study replicated, therefore Experiment 1 is a close replication of Exp. 2 of Bartsch and Shepherdson (2022). The goal of that previous study was to investigate whether maintenance in WM can be replaced by relying on information stored in episodic LTM, thereby freeing capacity for additional information in WM. Participants encoded word pairs into LTM, and then completed a WM task, also involving word pairs. Crucially, the pairs presented in each WM trial comprised varying numbers of new pairs and the previously learned LTM pairs. In the critical Exp. 2 of Bartsch and Shepherdson (2022), which we aim to replicate here, we orthogonally manipulated the number of new and LTM pairs used in the WM task. When WM load was low, performance declined with the addition of LTM pairs, but remained superior to performance with the matched set size comprising only new pairs. By contrast, when WM load was higher, adding LTM pairs did not negatively affect performance.

In Experiment 2 we introduced another “WM pure” condition, with trials of four to-be-remembered pairs always consisting of new pairings only. If the cost of adding stimuli for which participants have prior knowledge at a small set size was truly caused by participants not having to decide of whether to rely on WM or LTM, then WM performance in a similar condition at higher overall set size (i.e., the 4 + 2 condition) should have deteriorated compared to the new “WM pure” condition. This was not the case, leading to Experiments 3 and 4, in which we assessed whether there are indeed conditions in which only WM contributes to performance as opposed to the view that there is *always* a contribution of LTM. Here, we built on the assumption that under conditions of a contribution from LTM, these LTM traces of memoranda could benefit or hamper performance in WM tasks depending on the match between the traces stored in LTM and the ones to-be stored in WM in the current trial, yielding proactive facilitation and proactive interference, respectively. In line with the hypothesis that there are indeed conditions in which *only* WM contributes to performance, we showed that any proactive benefit or interference of knowledge from LTM was absent at set sizes that are sub-capacity.

## Experiment 1

**Figure 1:**
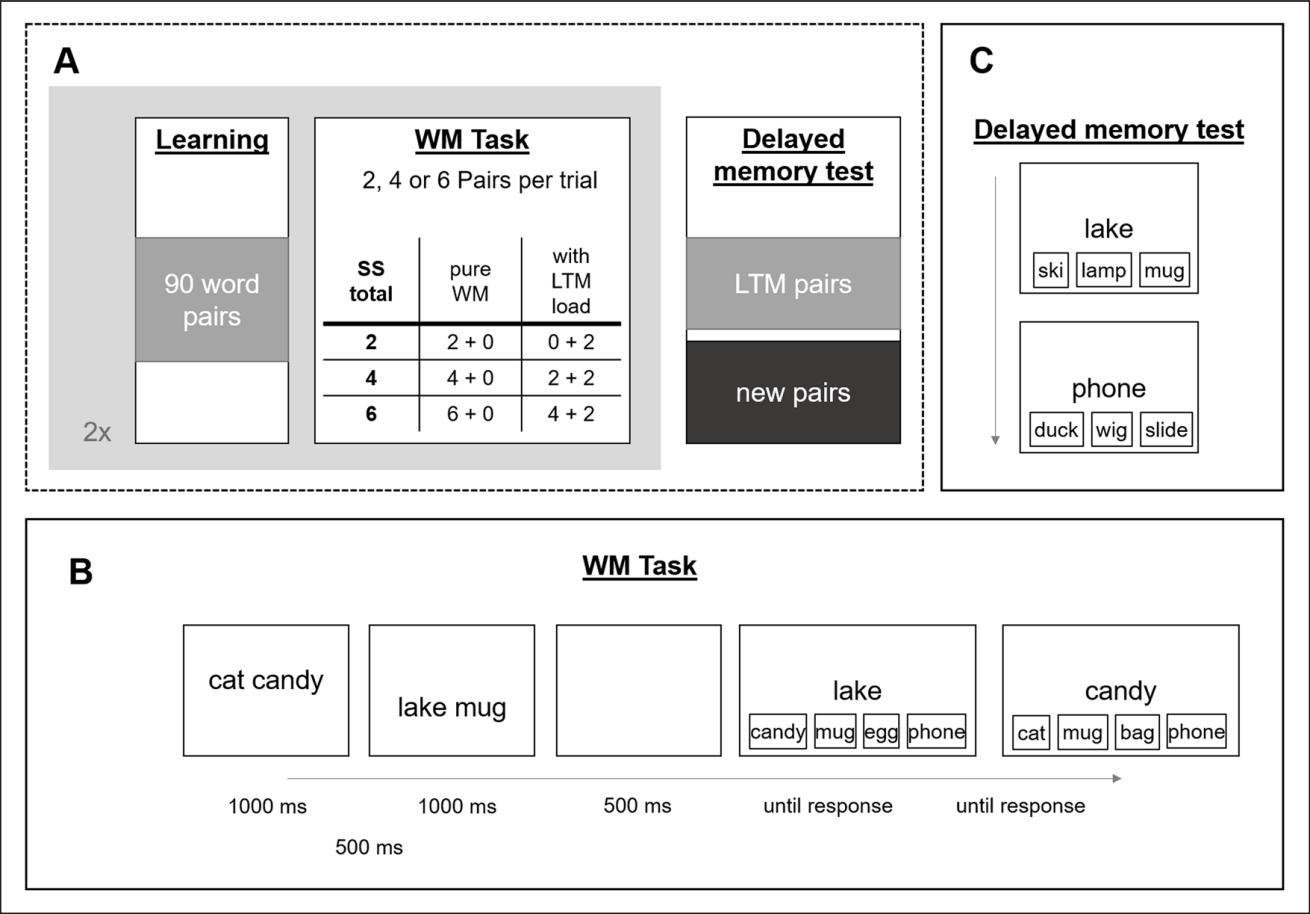
General procedure of Experiment 1 **(A)**, with the WM task **(B)** and the Delayed memory test **(C)**.

In Experiment 1 we aimed to assess the robustness of the pattern of results obtained in a previous study, showing that when WM load was low, memory for new pairs deteriorated with the addition of stimuli for which participants had prior knowledge but remained superior to performance with the matched set size comprising only new pairs. By contrast, when WM load was higher, adding stimuli for which participants had prior knowledge did not negatively affect memory for the new pairs. We followed the same procedure as in Experiment 2 of Bartsch and Shepherdson (2022), involving an LTM learning phase followed by WM trials; in which we independently varied WM and LTM load. In doing so, we aimed to assess the extent to which WM performance is impervious to changes in LTM load, and if so whether this is consistent across different levels of WM load. To this end we created six conditions, three including only new pairings (*2 + 0, 4 + 0*, and *6 + 0* condition) and three also including LTM pairs (*0 + 2, 2 + 2*, and *4 + 2* condition), leading to trials with the total memory load of 2, 4 or 6 pairs (see Figure 1A).

### Method

#### Participants

We collected data from 28 participants (M_age_ = 22.17 years) in the lab. All participants’ first language was German or Swiss-German, and they reported normal or corrected-to-normal vision. Participants signed an informed consent form prior to the study and were debriefed at the end. This and the following experiments were approved by the ethics committee of the philosophical faculty of the University of Zurich (Nr. 21.12.1). We chose the sample size for this, the second and fourth experiment because it was sufficient to detect the effects of interest in the original study (see (Bartsch & Shepherdson, 2022). The use of Bayesian statistics means that the sample size could have been increased in case of ambiguous evidence (Rouder, 2014).

#### Materials and procedure

Figure 1 provides an overview of the general procedure of Experiment 1. The experiment is a close replication of Exp 2 of Bartsch and Shepherdson (2022), with the difference being the inclusion of the 0 + 2 condition and dropping all conditions with LTM load of 4. As in the original, this experiment and consisted of 3 phases: an LTM learning phase in which subjects were simply presented with 90 pairs of concrete words, a subsequent WM task phase in which either 2, 4 or 6 word-pairs were presented sequentially from the top to the bottom of the screen, and a final delayed memory test for the pairs presented in the previous two phases. Performance on this final delayed memory test was used as an index of successful learning.

Stimuli consisted of random pairs of 1490 concrete German nouns with an average normalized Lemma frequency of 40.26. As in a previous study, the learning phase simply entailed the presentation of the material to the participant for 4000 ms, with an inter-stimulus-interval (ISI) of 1000 ms, with no test prior to the WM phase. The WM task was an immediate memory test in which participants needed to remember arbitrary word pairs (e.g., cat–candy, lake–mug), presented to them sequentially for 1000 ms each, separated by a 500 ms ISI, and were tested with a four-alternative forced choice procedure. On each test, one word from a pair was presented as a memory cue, and participants had to choose one out of four response options by button press: the target (previously paired with the cue), another item which was paired with a different word in the same trial (within-trial intrusion probe), a word that was learned within a different pair in the learning phase (LTM lure), or a new item. With this type of task, we ensure that the search set at test can specifically measure binding memory – rather than a familiarity signal for items – representing the core of WM functioning (for similar approaches and details see Bartsch & Shepherdson, 2022, 2023; Wilhelm et al., 2013).

Here, the WM trials included overall set sizes of 2, 4, or 6 pairs, that either consisted of pure WM trials (*2 + 0, 4 + 0*, and *6 + 0* condition), or included two previously learned LTM pairs (*0 + 2, 2 + 2*, and *4 + 2* condition). We relied on participants noticing that in set size 4 trials, none of the pairs were pre-learnt, in order to keep it as close to the original set-up as possible. The set size of a trial was predictable as the pairs were presented sequentially from the top to the bottom of the screen, with the first presented pair at larger set sizes being presented closer to the top of the screen than for smaller set sizes (e.g., set size 2). All pairs presented in each WM trial were tested in random order. In trials including LTM pairs, the within-trial lure randomly came from a within-trial LTM pair or a new pair. Following this first iteration (first mega-block) of the LTM learning and the WM task, subjects underwent a second LTM learning phase for another 90 word-pairs, followed yet again by a WM task (second iteration = second mega-block), both of which were procedurally identical to the first instances of each. The final part of the study consisted of a delayed memory test for all the pairs (both new and LTM) used in the experiment. Here, subjects were presented with the same four-alternative forced choice procedure for every pair of the experiment again.

The two-hour experiment comprised 90 WM trials in total, with 15 trials for each combination of WM load and LTM load, equally distributed across the 15 blocks. Within a block of 6 trials, the trials per cell of the design occurred in random order.

#### Data Analysis

##### WM task performance

The data from the WM task were analysed using Bayesian generalized linear mixed models (BGLMM) implemented in the R package *brms* (Bürkner, 2017, 2018). The dependent variable was the proportion of correct responses relative to the number of retrievals for each cell of the design. Correct responses were defined as recalling the target item from the four alternatives (target, within-trial intrusion, LTM lure, or new item). Therefore, we assumed a Binomial data distribution predicted by a linear model through a logit link function (i.e., an aggregated logistic regression). Apart from ensuring that predicated probabilities for correct responses are in the range between 0 and 1, the logit link function also increases the power to detect effects close to ceiling or floor performance, due to its non-linear transformation of probabilities to the parameter space (Dixon, 2008; Šimkovic & Träuble, 2019). The fixed-effects were WM load (0, 2, 4) and LTM load (0, 2) as well as their interaction. Following the recommendation of Barr and colleagues (Barr, Levy, Scheepers, & Tily, 2013; see also Schielzeth & Forstmeier, 2009) we implemented the maximal random-effects structure justified by the design: by-participant random-intercepts and by-participant random-slopes.

For regression coefficients, we used moderately informative Normal priors with a standard deviation of 1. These define a default prior analogous to that proposed by Rouder et al. (2012) for Gaussian General Linear Model. We calculated Bayes Factors (BFs) using the Savage-Dickey density ratio to estimate the strength of evidence for our specific hypotheses (Wagenmakers et al., 2010). Hereby, the Bayes factor is equal to the ratio of the prior density and posterior density of the test-relevant parameter at a given constraint. For instance, with the BF we can calculate the evidence for the effect of WM load (BF_10_) by calculating the ratio of posterior density to prior density at the value zero. When testing directed hypotheses, we calculated Bayes Factors as the ratio of the posterior density in the expected direction (e.g., larger than zero) over the other direction (e.g., smaller than zero).

Bayes Factors larger than 1 give evidence for an effect (i.e., in favor of a difference ≠ 0), whereas a BF_10_ lower than 1 provides evidence against an effect and hence evidence for the null hypothesis. We considered BFs > 3 as substantial evidence for one hypothesis over the other, and regarded BFs < 3 as inconclusive (Kass & Raftery, 1995).

We estimated posteriors of the full models using an MCMC algorithm (implemented in Stan; Carpenter et al., 2017) that estimates the posteriors by sampling parameter values proportional to the product of prior and likelihood. These samples were generated through 4 independent Markov chains, with 2000 warmup samples each, followed by 3000 samples drawn from the posterior distribution which are retained for analysis. Following Gelman and colleagues (2013), we confirmed that the 4 chains converged to the same posterior distribution by verifying that the R-hat statistic – reflecting the ratio of between-chain variance to within-chain variance – was < 1.01 for all parameters, and we visually inspected the chains for convergence.

##### LTM task performance

As with the WM task, we analysed the data from the LTM task using Bayesian generalized linear mixed models (BGLMM) implemented in the R package *brms* (Bürkner, 2017, 2018). If not stated below, any details of the analysis were conducted exactly as for the data of the WM task. Correct responses were defined as recalling the target item from the lure alternatives (within-trial intrusion, LTM lure, or new item). Here, the fixed-effects were WM trial set size (2, 4 vs. 6), and pair type (LTM vs. WM pair) as well as their interaction. Our primary motivation in this analysis was to assess the extent to which participants remember the LTM pairs better than the WM pairs, making this essentially a check of our LTM manipulation.

### Results

The data and the analysis scripts of this and all following Experiments can be accessed on the Open Science Framework (https://osf.io/spkhb).

#### WM task performance

**Figure 2:**
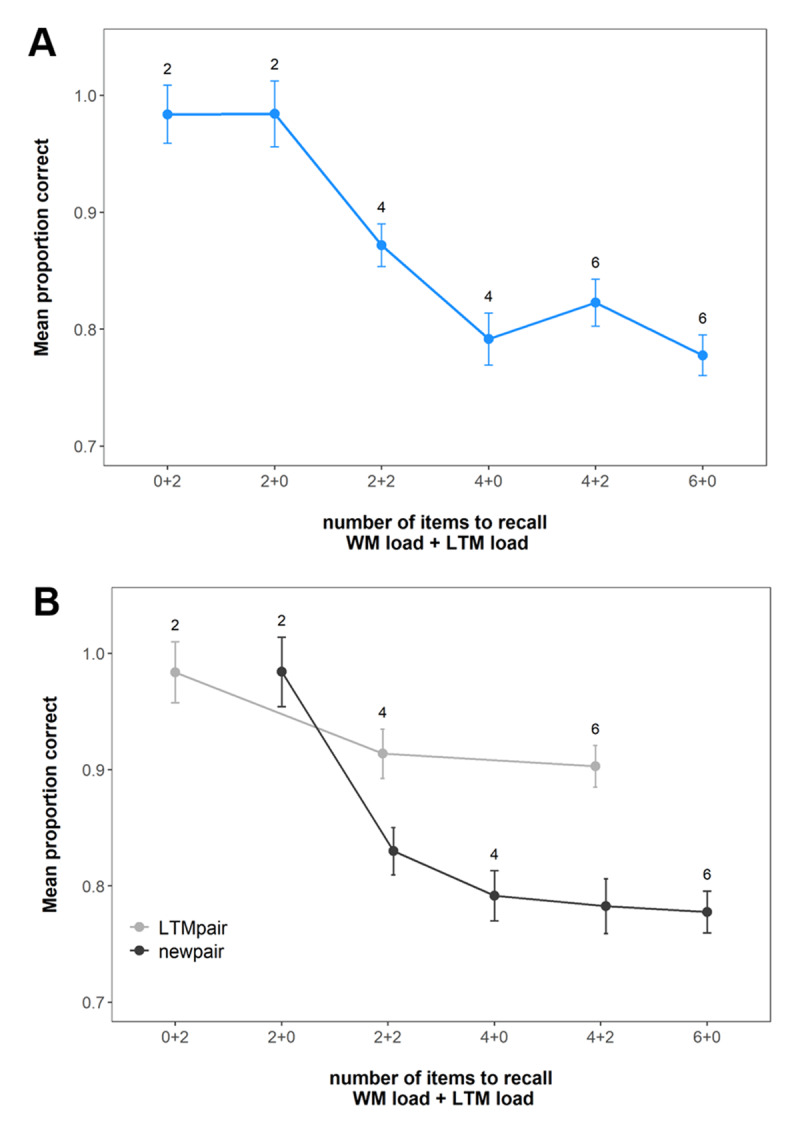
Mean immediate recall performance in Experiment 1. **(A)** shows the performance across all pairs at the set sizes. **(B)** shows the performance for the LTM and new pairs separately. The numbers in the figures show the total set size of the respective conditions. Error bars represent within-subject confidence intervals.

Figure 2 shows the mean probability of choosing the correct response option across the conditions. Our first question was whether we could replicate the previous studies’ pattern of results – namely a drop in performance when LTM pairs were added at low WM load (two pairs) and the lack of such a drop at higher WM load (four pairs). In this case, our answer was a clear “yes”. Specifically, performance in conditions with both LTM and WM pairs was always superior to performance in matched set size conditions with new pairs only; however, performance also deteriorated with increasing LTM load when WM load was low (LTM load 0 vs. 2 at WM load of two pairs: BF_10_ = 1.39 × 10^5^). Nonetheless, LTM load did not affect recall performance at higher WM load (four pairs; BF_10_ = 0.75), if anything performance improved with LTM load (LTM load 0 vs. 2 > 0: BF_10_ = 30.41), providing evidence that individuals can outsource workload to LTM to optimise performance when needed. Pairwise comparisons revealed that memory performance was better in LTM-available conditions relative to matched set-size trials without or with fewer LTM pairs (WM load 4 vs. WM load 2 + LTM load 2; BF_10_ = 1.46 × 10^19^ and WM load 6 vs. WM load 4 + LTM load 2; BF_10_ = 8.14 × 10^15^).

#### LTM task performance

**Figure 3:**
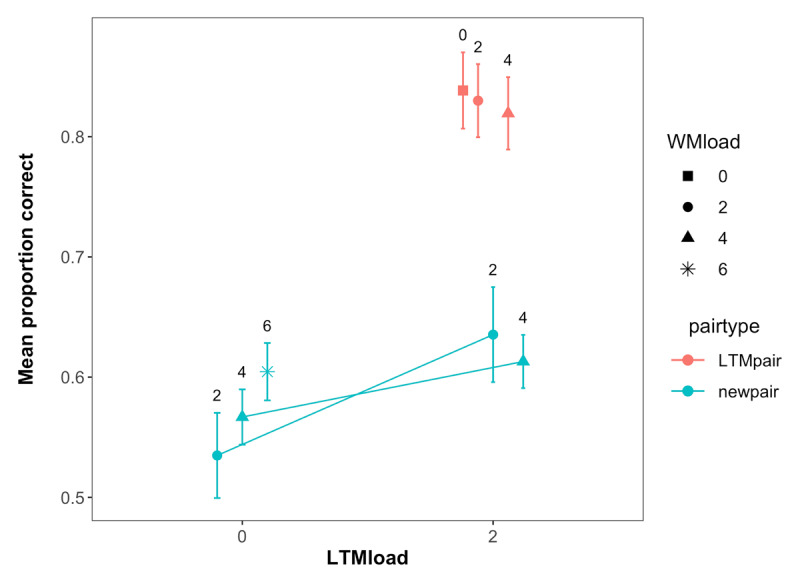
Mean performance in the LTM task across WM load, LTM load and pair type in Experiment 1. Labels represent the WM load. Error bars represent within-subject confidence intervals.

Performance in the LTM test for all the pairs presented across the experiment is shown in Figure 3. Our primary motivation for the LTM test was to assess the extent to which participants remembered the LTM pairs better than the new pairs, making this essentially a check of our LTM manipulation. As can be seen in Figure 3 and supported by a main effect of pair type in our analysis (BF_10_ = 3.11 × 10^19^), subjects had better memory for the trained LTM pairs, indicating that the manipulation was successful.

### Discussion

Experiment 1 replicated previous findings and showed that participants benefit from prior knowledge stored in episodic LTM in WM tasks, yet that this benefit seems to be limited to situations of high working memory load. Although this speaks to the benefit of LTM to WM being specific to situations in which some negotiation between WM and LTM already is taking place (e.g. at higher WM load; *WM overload hypothesis*), there is an alternative explanation: The cost of the addition of LTM pairs at low WM load, could be due to the ambiguity about whether a decision has to be made to recruit LTM or not at set size 4 (*Decision ambiguity hypothesis*). Specifically, the conclusion that adding LTM pairs negatively affects memory performance for new pairs is based on a comparison between the 2 + 0 and 2 + 2 conditions, with memory for new pairs superior in the former. Because set size in any trial was detectable when presentation began, and since sets of size 2 never involved a mixture of new and pre-learnt pairs (i.e., they consisted of all new or all pre-learnt pairs), these small sets never required a participant to determine, pair-by-pair, whether LTM could be relied on for memory of that pairing. By contrast, sets of size 4 could either consist of new pairs only, or a mixture of new and pre-learnt pairs. This set size may thus have required participants to make rapid judgements about whether each pair had been learnt before (and thus could be offloaded to LTM), or was novel. The inferior performance for new pairs in the 2 + 2 condition may thus be attributable to greater decisional ambiguity in this condition, relative to the 2 + 0 condition we compared it to. In order to adjudicate between these two hypotheses, we ran a second Experiment.

## Experiment 2

In Experiment 2 we aimed to determine whether the apparent cost of adding stimuli for which participants have prior knowledge at a small set size was in fact caused by differences across conditions in ambiguity about whether they would have to decide to rely on WM or LTM for each pair’s retention (*Decision ambiguity hypothesis*) – and not because at set size 2 only WM contributes to performance (*WM overload hypothesis*). To achieve this, we introduced a “non-ambiguity” condition at set size 4, with trials of four to-be-remembered pairs always consisting of new pairings only. In other words, upon the presentation of the first pair of a trial with set size 4, participants could correctly conclude that none of the pairs in the trial would have been pre-learnt, and thus that there was no need to determine which pairs needed to be stored in WM, and which could be “offloaded” to LTM (Bartsch & Shepherdson, 2023).

Equivalent to Experiment 1, after an initial LTM learning phase, the WM task tested memory for word pairs at varying set sizes. As shown in Figure 1, these trials included 2, 4 or 6 pairs, which *could* partly be known to the participant from the LTM learning phase (i.e., in conditions 0 + 2 and 4 + 2). Critically, trials with SS4 always consisted of new pairings only. If the cost of adding stimuli for which participants have prior knowledge at a small set size is truly driven by the memory load being sub-capacity, then adding two LTM pairs to a a “non-ambiguity” set size of four pairs should result in equivalent performance (4 + 0 == 4 + 2), just as shown in Experiment 1 and a previous study (*WM overload hypothesis*; Bartsch & Shepherdson, 2022). If, instead, the previous pattern of results was caused by participants not having to decide whether to rely on WM or LTM when set size was 2 (but having to make such decisions in all other conditions), then WM performance in the 4 + 2 condition should drop compared to the “non-ambiguity” condition of 4 + 0 (*Decision ambiguity hypothesis*).

### Method

#### Participants

We collected data from 29 participants (M_age_ = 20.97 years) in the lab. All participants’ first language was German or Swiss-German, and they reported normal or corrected-to-normal vision. Participants signed an informed consent form prior to the study and were debriefed at the end.

#### Materials and procedure

Experiment 2 was consistent with the Materials and Procedure used in Experiment 1, except from the single change of dropping the 2 + 2 condition, so that all set size 4 trials were “pure WM trials”, in which participants experienced no ambiguity about the decision concerning whether to rely on WM or LTM for each pair’s retention.

#### Data Analysis

The data from the WM and LTM task were analysed equivalently to Experiment 1.

### Results

#### WM task performance

**Figure 4:**
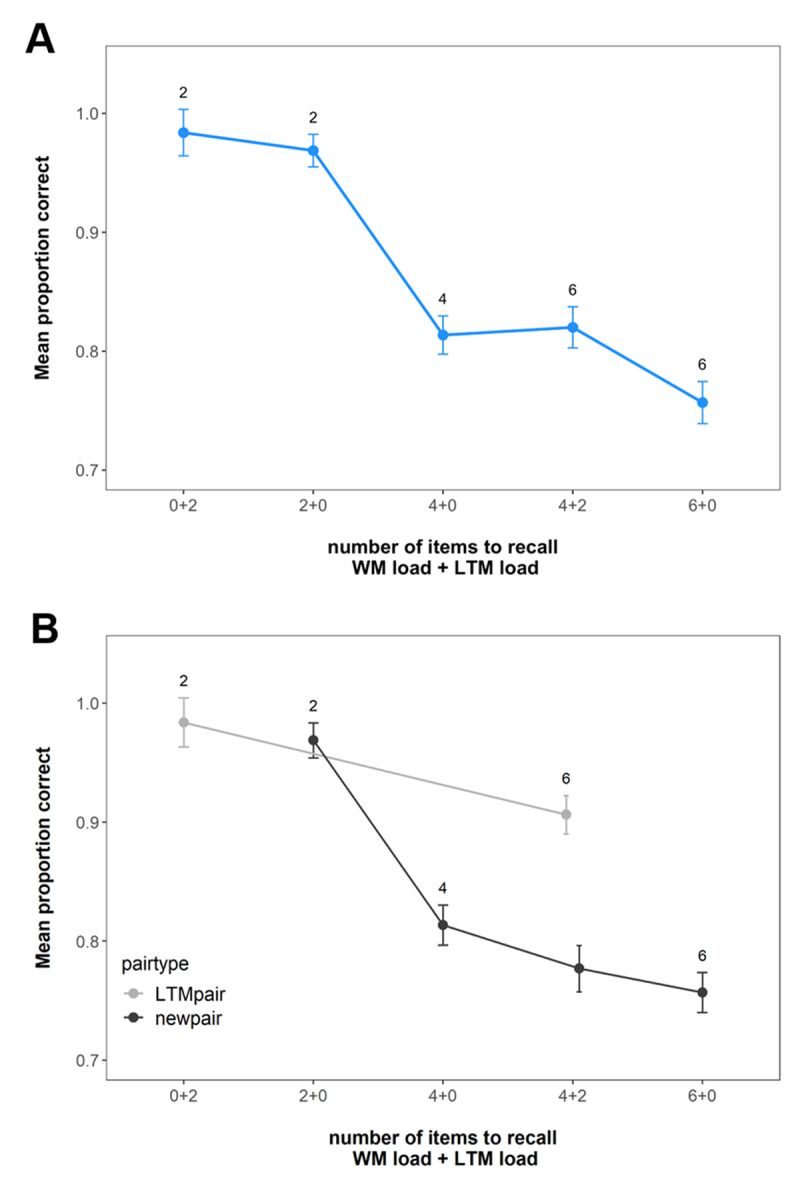
Mean immediate recall performance in Experiment 2. **(A)** shows the performance across all pairs at the set sizes. **(B)** shows the performance for the LTM and new pairs separately. Error bars represent within-subject confidence intervals.

Figure 4 shows the mean probability of choosing the correct response option across the conditions. Our first question was whether adding two LTM pairs to a “non-ambiguity” set size of four pairs would result in equivalent performance (4 + 0 == 4 + 2), just as shown in Experiment 1 and a previous study (Bartsch & Shepherdson 2022) – or whether WM performance in the 4 +2 condition would drop compared to the “non-ambiguity” condition of 4 + 0. As can be seen in Figure 4A and supported by the BGLMM, LTM load did not affect overall recall performance at higher WM load (four pairs; BF_10_ = 0.08), providing evidence against the *Decision ambiguity* hypothesis that benefits of prior knowledge are specific to conditions in which there is no ambiguity concerning decisions about using WM or LTM. Pairwise comparisons replicated the previous finding that memory performance was better in LTM-available conditions relative to matched set-size trials without LTM pairs (WM load 6 vs. WM load 4 + LTM load 2; BF_10_ = 6.62 × 10^16^).

#### LTM task performance

**Figure 5:**
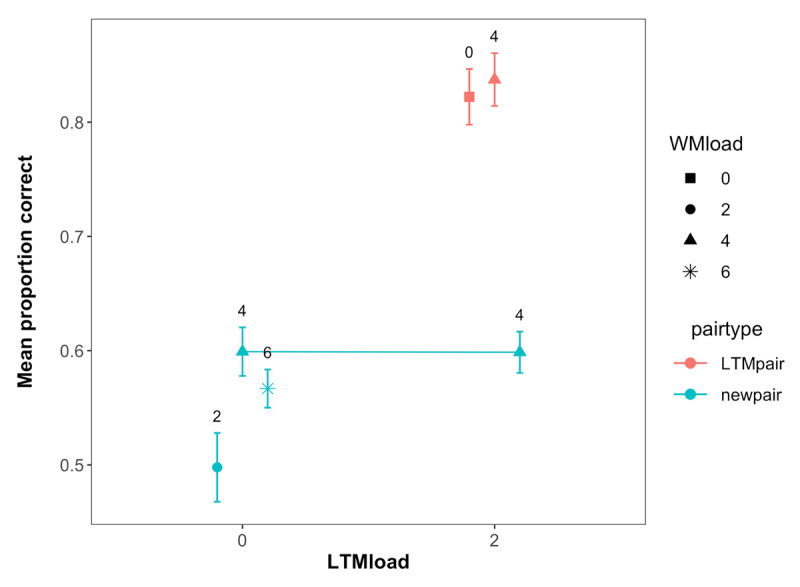
Mean performance in the LTM task across WM load, LTM load and pair type in Experiment 2. Labels represent the WM load. Error bars represent within-subject confidence intervals.

Performance in the LTM test for all the pairs presented across the experiment is shown in Figure 5. Our primary motivation for the LTM test was again to assess the extent to which participants remembered the LTM pairs better than the new pairs, making this essentially a check of our LTM manipulation. As can be seen in Figure 5 and supported by a main effect of pair type in our analysis (BF_10_ = 7.57 × 10^21^), subjects had better memory for the trained LTM pairs, indicating that the manipulation was successful.

### Discussion

Experiment 2 showed that although we introduced another “non-ambiguity” condition, with trials of four to-be-remembered pairs always consisting of new pairings only – and participants not experiencing any ambiguity regarding whether to rely on WM or LTM at set size 4 – performance did not drop at higher WM load when adding previously learned pairs. This result speaks in favor of the *WM overload* hypothesis that the benefit of prior knowledge to WM happens only when some negotiation of information between WM and LTM is already necessary. Yet, there is still no direct evidence whether there are indeed conditions in which only WM contributes to performance, without any contribution from episodic LTM (*WM overload* hypothesis, see Table 1). Specifically, our conclusion that WM is shielded from LTM at low loads is open to another alternative explanation: It is possible that any benefit from LTM would be undetectable in measures of performance in cases were the use of WM alone would result in the same level of performance.^2^ In other words, WM would still be able to benefit from LTM without a noticeable impact on performance. This alternative explanation would also predict that the benefit of LTM would become observable only when the memory load exceeds WM capacity, for which WM alone cannot handle the task well. This logic is supported by recent work showing that different involvements of WM to represent LTM-available information, as recorded in the EEG, may not influence accuracy at load 1 (Ozdemir et al., 2024; Yucel et al., 2023).

To provide a stronger test for the WM overload hypothesis against the *Obligatory LTM* hypothesis, we conducted Experiments 3 and 4, in which we investigated whether there is an influence of LTM in form of proactive facilitation and interference present irrespective of WM load. Proactive interference effects allow us to also test the above alternative explanation, as they circumvent the problem of potentially equally good performance at low memory load, since any contributions from LTM in interference cases would negatively affect performance only in the case that WM performance actually is influenced by LTM.

## Experiment 3

In Experiment 3 we aimed to assess whether there are indeed conditions in which only WM contributes to performance (*WM overload hypothesis*) as opposed to the view that there is *always* a contribution of LTM (*Obligatory LTM hypothesis*). To achieve this, we built on the assumption that under conditions of a contribution from LTM, these LTM traces of memoranda could either benefit or hamper performance in WM tasks depending on the match between the traces stored in LTM and the ones to-be stored in WM in the current trial, yielding proactive facilitation (PF) and proactive interference (PI), respectively. PI refers to detrimental effects of previously learned material on memory acquired later, and it has been shown that information that is stored and retrieved from LTM is strongly affected by PI (Crowder, 1976). In contrast, WM representations have been shown to be immune to proactive interference (e.g., Cowan et al., 2005; Oberauer et al., 2017; Oberauer & Bartsch, 2023).

Equivalent to Experiment 1 and 2, after an initial LTM learning phase, the WM task involved a test of memory for word pairs at varying set sizes. Crucially, sets in the WM task consisted of new pairings (new-new), original pairings from the LTM-learning phase (old-match), and a mismatched pairing of a singleton from the LTM-learning phase (e.g., a word) to a new paired associate (e.g., a new word; old-mismatch). If there are indeed conditions in which only WM contributes to performance (as predicted by the WM overload hypothesis), then any proactive facilitation or interference of knowledge from LTM should be absent at small set sizes that are sub-capacity. If instead the Obligatory LTM hypothesis holds (see Table 1), and participants always use LTM to improve performance in the WM test, they should benefit from it in the old-match pairings but will show costs for the old-mismatch pairings relative to new pairings – regardless of set size.

### Method

#### Participants

We collected data from 142 participants online via Prolific (M_age_ = 28.57 years). We chose the sample size for this experiment because (1) we ran fewer trials per participants online than in the in-person Experiments 1 and 2 and compensated by testing higher Ns (Rouder & Haaf, 2018) and (2) this sample size was sufficient to detect similar effects of interest in a previous study, which was also run on Prolific (see Bartsch & Shepherdson, 2022). We excluded 21 participants as their response accuracy in the delayed memory test was below chance performance and 1 participant as their overall response accuracy across all conditions was greater than 2 standard deviations below the overall mean. Therefore, the final sample included 120 participants. All participants’ first language was English. Participants gave informed consent prior to the study and were debriefed at the end.

#### Materials and procedure

As in the previous two experiments, participants in Experiment 3 underwent an LTM learning phase, in which episodic LTM of 54 word pairs (e.g., dog – pony) was built up. Their memory for those pairs was tested afterwards with a three-AFC procedure – including the target, an LTM lure and a new item as response options. Participants received feedback about their response and were shown the correct option, in case they had made a mistake. Performance was used as an index of successful learning.

Subsequently, participants completed trials of a WM task, also involving word pairs. Crucially, the memory set presented in WM trials of varying set size (2, 3, and 4) consisted of new pairings (new-new), original pairings from the LTM-learning phase (old-match), and a mismatched pairing of a singleton from the LTM-learning phase (e.g., dog) to a new paired associate (e.g., glass; old-mismatch). Importantly, in case of the old-mismatch stimuli, at test, the *LTM lure* response option always was the word from the original pairing (e.g., pony). Stimuli consisted of random pairs of 360 concrete English nouns. The 30-minute experimental session comprised 27 WM trials in total, with 3 trials per set size (see Table 2), equally distributed across 3 blocks. Within a block of 9 trials, the trials per cell of the design occurred in random order. Data were collected online via Prolific.

**Table 2:** The composition of trials at varying set sizes (SS) of Experiment 3 and 4.


SS	NEW – NEW	OLD – NEW (MISMATCH)	OLD – OLD (MATCH)

**2**	x	x	

		x	x

	x		x

**3**	x	x	x

**4**	2x	x	x

	x	2x	x

	x	x	2x


*Note*: Rows represent the possible compositions at each of the set sizes. For instance, at set size 2, trials consisted of either **(A)** one new-new and one mismatch pair; **(B)** one mismatch pair and one match pair; or **(C)** one new-new and one match pair.

#### Data Analysis

We analysed the data similarly to Experiments 1 and 2, with the following changes: Here, the fixed-effects were set size (2, 3, 4) and pair type (new-new, old-match, old-mismatch) as well as their interaction. Using the *hypothesis* function of the *brms* package (Bürkner, 2017) in R, we tested the evidence for proactive facilitation and interference effects at each set size.

### Results

We first ensured that subjects had built-up LTM for the pre-learnt pairs. Results showed that subjects correctly responded to 70.8% (SD = 17.3) of pairs in the final test (where chance performance on the 4AFC is 25% correct).

**Figure 6:**
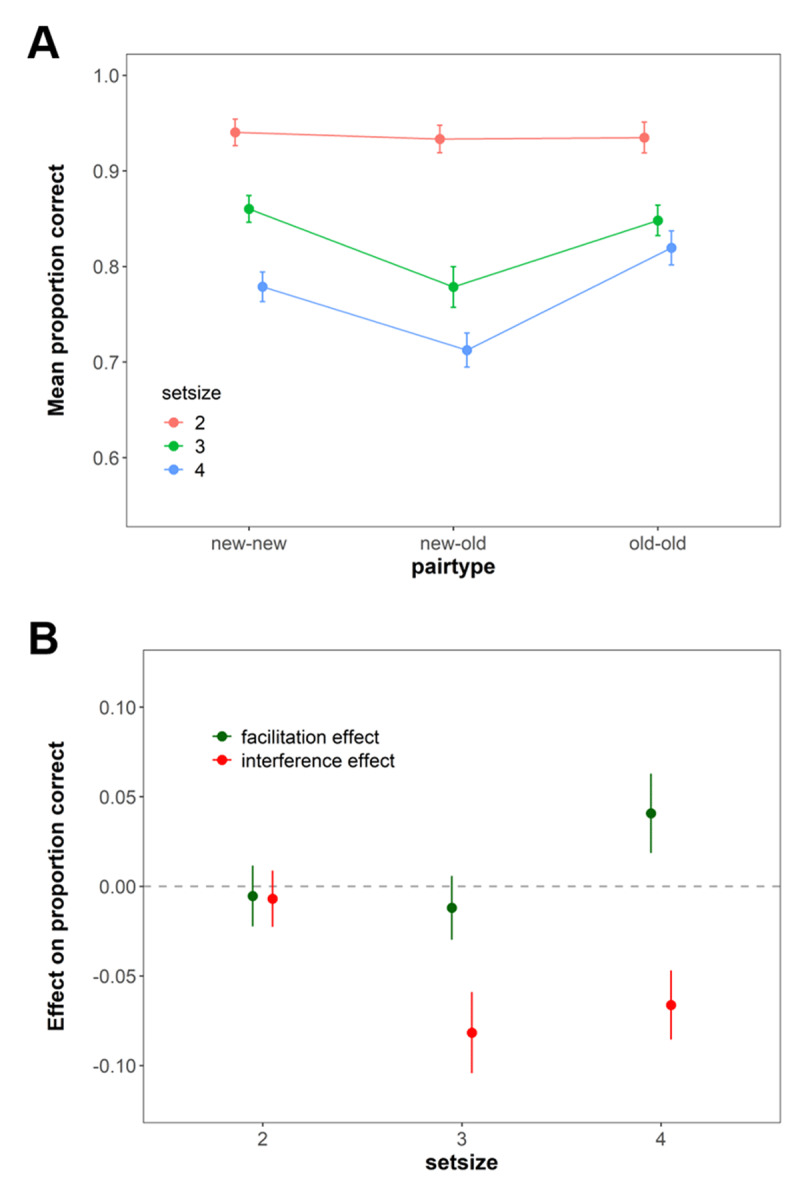
Mean immediate recall performance in Experiment 3. **(A)** shows the performance across all pairs at the set sizes. **(B)** shows the proactive facilitation (difference between old-old and new-new pairs) and interference effects (difference between. old-new and new-new pairs). Error bars represent within-subject confidence intervals.

The performance in the WM task is shown in Figure 6. Here we wanted to test the hypothesis that if subjects rely exclusively on WM for sub-capacity set sizes (e.g., SS = 2), then they should neither show a cost in performance for mismatch pairings, nor a benefit for match pairings – as these effects reflect a contribution of LTM. The results revealed evidence in favour of this hypothesis. Specifically, we found evidence against any effects of whether information matched the pairing stored in LTM or not at SS2 (difference of new-new vs. old-old pairs: BF_01_ = 3.53 and difference of new-new vs. new-old pairs BF_01_ = 7.81, respectively). By contrast, at SS3 and SS4 we found evidence for proactive interference effects (BF_10_ = 6.14 × 10^17^ and BF_10_ = 4.17 × 10^33^, respectively) as well as facilitation effects at SS4 (BF_10_ = 2.91 × 10^15^).

### Discussion

With Experiment 3, we directly tested whether the enhancement of performance is limited to situations where some negotiation of information between WM and LTM is already necessary (*WM overload* hypothesis) or whether there is always a contribution of LTM (*obligatory LTM* hypothesis). In line with the former, we showed that with performance at SS2 we indeed isolated a condition in which only WM contributes to performance. It speaks against the *obligatory LTM* hypothesis that there is always a contribution from episodic LTM. The absent proactive interference effect at low WM load further speaks against the alternative explanation that WM benefits from LTM at low loads without a significant impact on behavioral performance. If WM were to draw on LTM at low as well as high WM loads, performance should have deteriorated in the presence of mismatch pairs.

## Experiment 4

The goal of Experiment 4 was to replicate and generalize the findings of Experiment 3 using visual stimuli. This has the further advantage that including visual stimuli allows for a continuous error measure as dependent variable, which is commonly used in visual WM research and does not produce ceiling effects even at set size 1 (e.g., Wilken & Ma, 2004).

### Method

#### Participants

We collected data from 33 participants (M_age_ = 22.21 years) in the lab. We excluded 2 participants as their response accuracy in the final delayed memory test at the end of the learning phase was at chance performance. Therefore, the final sample included 31 participants. All participants’ first language was German or Swiss-German, and they reported normal or corrected-to-normal vision. Participants signed an informed consent form prior to the study and were debriefed at the end.

#### Materials and procedure

**Figure 7:**
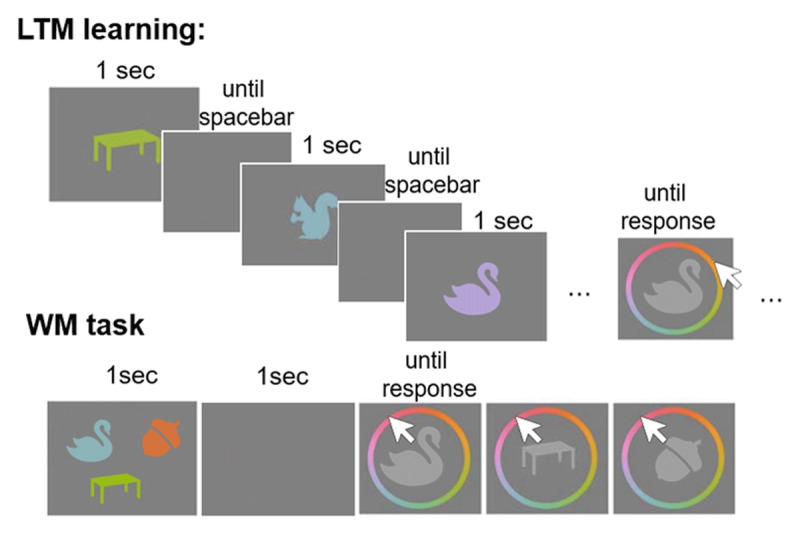
General procedure of Experiment 4 showing the LTM learning phase with the continuous reproduction test; and the flow of events in the WM test, here a set size 3 trial is depicted.

Experiment 4 follows the same logic and paradigm as Experiment 3, except that here, the stimuli were color-object conjunctions (see Figure 7). We used 389 silhouette images of concrete nameable objects that were used in a previous study (Oberauer et al., 2017). These were presented in conjunction with one of 360 colors from a color wheel in the CIE L × a × b color space, centered on L = 70, a = 20, and b = 38, with a radius of 60. This is the color wheel most commonly used in continuous-reproduction tests of visual WM, because it consists of colors that are approximately equidistant in psychological similarity space, and approximately equally bright (for a critical discussion see Bae et al., 2014). The images were presented uniformly in the chosen color against a gray background.

As in Experiment 3, the experiment consisted of two phases: A learning phase and a WM task. The procedure further closely followed previous implementations (Oberauer et al., 2017) and builds on findings of Brady et al. (2013) who showed that people can learn large numbers of object-color conjunctions well.

The learning phase entailed the sequential presentation of groups of 10 colored objects, followed by a test of these 10 objects in random order using a continuous reproduction task. In this task a color wheel was presented around the object, shown in a lighter gray, rotated into a random orientation from trial to trial, and a mouse arrow appeared in the object’s center (see Figure 7). Once participants moved the mouse away from the center in the direction of one of the colors of the color wheel, the object assumed that color. Thus, by moving the mouse the participants continuously adapted the object’s color. They were instructed to reproduce the color they remembered for the object as accurately as possible. They submitted their response by a mouse click. Participants received feedback about their response, by being shown the degree of deviation from the true color. Further, the object was presented to them in the correct color for 1 sec. We interspersed study phases with test phases to capitalize on the testing effect, in order to further boost long-term learning (Roediger III & Karpicke, 2006). After being presented with and tested on 120 color-object conjunctions, participants performed a final delayed memory test for all these LTM color-object conjunctions in random order.

In the WM task participants were presented with 9 blocks of 9 trials each, consisting of one of each of the possible compositions of set size and pair type (see Table 2). Old-Old objects consisted of objects that were presented in the color learned in the LTM learning phase. Old-New pairings consisted of one of the previously learned objects being presented in a new randomly chosen color. New-New pairings were never-seen-before objects paired with a color chosen at random. Across the first 7 blocks, sampling of objects was without replacement so that each of the 120 old objects was used exactly once, and in blocks 8 and 9, old objects from the earliest trials of the experiment were used a second time. New objects never repeated across the experiment.

#### Data Analysis

For this experiment, we analysed the response error defined as the deviation of the continuous response to the original colour an object was presented in. Specifically, we fit a Bayesian hierarchical mixture model dissociating responses resulting from a) retrieving the target item, b) erroneously retrieving the colour of another item presented in the trial (i.e., a swap or transposition error), c) erroneously retrieving the initially pre-learned colour for old-new pairs (i.e., transpositions/interference from LTM), and d) random guessing (Bays et al., 2009). We implemented this model in *brms* following the tutorial by Frischkorn and Popov (2023). In this model, we estimated the precision of memory representations (kappa) varying over set size (i.e., 2, 3, and 4).^3^ The probability of retrieving the memory item (P_M_), of committing a swap error (P_Swap_), or random guessing (P_G_) varied over set size and pair type. Following the example of Oberauer et al. (2017), we created a set of imaginary LTM colours for new-new and old-old pairs by randomly sampling values from a uniform distribution. As participants obviously did not actually see and associate these simulated colours with the objects in these conditions, the probability for LTM swaps should be close to zero in these conditions and served as a baseline for the probability of LTM swaps in the old-new conditions. But simulating these imaginary LTM colours allowed us to vary the probability of swaps from LTM (P_LTM_) over all set sizes and pairtypes.

For parameter estimation, we collected 2000 warmup samples followed by 3000 samples drawn from the posterior distribution that were retained for analysis from four MCMC chains. We confirmed convergence of the 4 MCMC chains by verifying that the R-hat statistic was smaller than 1.01 for all parameters.

With respect to the hypothesis, increases in the probability of recalling the target item for old-old pairs (i.e., pre-learned LTM pairs) compared to new-new pairs (i.e., WM pairs) would indicate facilitation of WM by LTM representations. Interference from LTM information with WM representation would result in decreases in the probability of recalling the target item for old-new pairs compared to new-new pairs, and an increased probability of transpositions from LTM compared to the randomly sampled LTM intrusions for new-new pairs.

### Results

Again, we first ensured that subjects had built-up LTM for the pre-learnt object-color pairs. As noted above, the results indicated that two subjects did not learn the object color associations (mean recall error of ~90°) and were thus discarded from the following analyses. The remaining 31 subjects had a mean recall error of 42.8° (SD = 12.3°) in the final memory test of the learning phase (where chance performance is at mean recall error of 90°) indicating that they successfully learned the object-color pairs.

**Figure 8:**
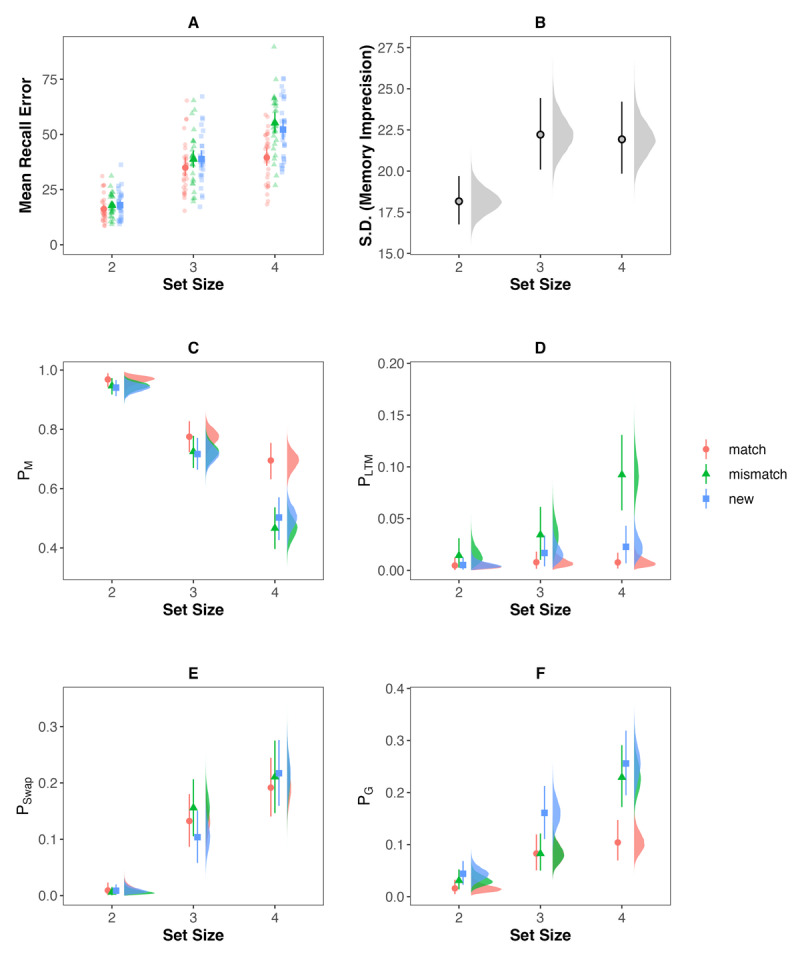
Descriptive plot of mean recall error **(A)** and estimated model parameters of the Bayesian hierarchical mixture modeling **(B–F)** of the behavioral performance in the immediate recall task of Experiment 4. The descriptive plot **(A)** illustrates the performance of all subjects (transparent points) as well as mean performance and the standard error. The plots of the model parameters show the full posterior distribution as well as the posteriors mean and 95% highest density interval.

Mean recall error in the WM task is shown in Figure 8A. Using a Bayesian Hierarchical Mixture Modelling approach, we tested if our findings from Experiment 3 – indicating that subjects only rely on WM for sub-capacity set sizes (e.g., SS = 2), whereas they rely on both WM and LTM for supra-capacity set sizes (e.g., SS = 4) – would generalize to visual material. As illustrated in Figure 8C, at set size 2 the probability of recalling the target item (P_M_) did not differ for object-colour pairs that matched the pairing stored in LTM and new object-colour pairs (BF_01_ = 3.72). Likewise, there was no cost on P_M_ for object-colour pairs that mismatched the pairs stored in LTM compared to new object-colour pairs at set size 2 (BF_01_ = 10.86). Thus, there was neither facilitation nor interference from LTM information with WM representations at set size 2. Results with respect to these differences were ambiguous at set size 3 (inconclusive evidence with respect to facilitation, BF_01_ = 1.78, but evidence against interference, BF_01_ = 5.55). At set size 4, however, there was credible evidence that WM benefitted from LTM information, as the probability of recalling the target item was credibly larger for object-colour pairs matching pairs stored in LTM then for new pairs (BF_01_ = 16.88). Yet, there was inconclusive evidence with respect to there being a cost in performance on P_M_ for pairs mismatching the pairs stored in LTM compared to new pairs (BF_01_ =2.02). Thus, on P_M_ we only found facilitation of WM performance by LTM, but no interference at set size 4.

Apart from evaluating costs on the probability of recalling the target item, our analyses also enable us to directly test if LTM intrusions were more likely across the different set sizes: The analyses of the probability of LTM intrusions (P_LTM_, see Figure 8D), revealed that LTM intrusions were equally likely for mismatch pairs compared to randomly generated LTM intrusions for new pairs,^4^ both for set size 2 (BF_01_ =17.38), and for set size 3 (BF_01_ = 8.32). Yet for set size 4, LTM intrusions occurred with a higher probability compared to the randomly generated LTM intrusions for new pairs, BF_10_ =10.16. In line with the results of the probability of recalling the correct item from memory (P_M_), this indicates that an exchange of information between LTM and WM only occurs at larger set sizes when the capacity of WM is reached or surpassed.

For completeness, Figures 8E/F illustrate the probability of swap errors to other colours also encoded in working memory and the probability of random guessing across set sizes and the different pair-type conditions. Integrating these probabilities with the above outlined results indicates that the improved memory recall when LTM information is matching WM pairs mainly reduces the probability of random guessing at set size 4. The increase in LTM intrusions at set size 4, however, was distributed across decreases in both the probability of recalling the target item and random guessing. This might explain why we did not find interference costs on the probability of recalling the target item at set size 4, although there was a credible proportion of LTM intrusions.

### Discussion

All in all, in Experiment 4 we replicated the findings from Experiment 3 and were able to generalize them to the visual domain: an exchange of information between LTM and WM occurs only when WM capacity is exceeded. Proactive interference effects were absent at low WM load, speaking against LTM representations influencing WM in these cases. As the continuous reproduction task is less prone to ceiling effects, these results also indicate that the lack of facilitation from LTM at smaller WM load cannot be explained by individuals already being at ceiling performance.

Nonetheless, the probability of retrieving an item from memory (P_M_) was still very high in the smallest set size in this experiment (P_M_ = 0.94; 95% HDI = [0.91; 0.97]). Yet, like the logistic link function we used in the binomial models in Experiments 1 to 3, the SoftMax link function used in the mixture models (for details, please see Frischkorn & Popov, 2023) similarly improves the power to detect effects close to ceiling or floor performance. In fact, although not credible, descriptively the probability of retrieving an item from memory was estimated to be slightly higher for old-old pairs compared to new-new pairs (Δ = 0.03; 95% HDI = [-0.01; 0.06]), indicating that there was the scope to detect facilitation effects even at set size 2.

Although we were able to generally replicate the finding that information exchange between LTM and WM is limited to situations in which WM capacity is reached, the specific pattern of interference and facilitation of WM performance differed between Experiment 3 and Experiment 4. Specifically, Experiment 3 showed strong interference effects and smaller facilitation, whereas Experiment 4 showed stronger facilitation and smaller interference effects. We turn back to this issue in the General Discussion.

## General Discussion

Previous research found that prior knowledge can boost WM storage when both novel and previously learnt word pairs must be retained on a short-term basis (Bartsch & Shepherdson, 2022, 2023). Still, it remained an open question whether there are indeed conditions in which only WM contributes to performance, as opposed to the view that there is *always* a contribution of LTM. Across four experiments we replicated these previous findings (Exp 1) and tested two alternative explanations (Exp 2, 3 and 4).

In Experiment 2 we showed that although we introduced another “no-ambiguity” condition, with trials of four to-be-remembered pairs always consisting of new pairings only – and participants not experiencing any ambiguity regarding whether they should rely on WM or LTM for each pair at set size 4 – performance did not drop at higher WM load when adding previously learned pairs. This result speaks for the original hypothesis that the benefit of prior knowledge to WM happens only when some negotiation of information between WM and LTM is already necessary.

With Experiments 3 and 4 we showed that for both verbal as well as visual material, information presented in trials of set size 2 was unaffected by proactive facilitation and interference, whereas performance was affected by these influences from LTM at set size 4. Under the assumption that when performance incorporates a contribution from LTM, these LTM traces of memoranda could benefit or hamper performance in WM tasks depending on the match between the traces stored in LTM and the ones to-be stored in WM in the current trial (i.e., yielding proactive facilitation and proactive interference, respectively), our results therefore speak for the *WM overload* hypothesis that there are indeed conditions in which *only* WM contributes to performance.

### The Contents of Working Memory are Shielded Against Proactive Interference

Our conclusions about conditions in which eLTM does *not* contribute to WM tasks – such as the word-pair binding task as well and the color-object conjunction task used here – are based on the assumption that the contents of WM are protected against proactive interference from earlier events that are no longer held in WM but are still represented in episodic memory. This assumption has a longstanding history, originating from research conducted decades ago (Conway & Engle, 1994; Craik & Birtwistle, 1971; Davelaar et al., 2005; Unsworth & Engle, 2007b; Wickens et al., 1963). Recent studies have extended evidence for the assumption that WM contents are immune to interference from eLTM. These studies consistently demonstrate that recall from WM remains unaffected by proactive interference from previous trials, as long as the memory load remains within the estimated capacity of WM (Bartsch & Oberauer, 2023; Oberauer et al., 2017; Oberauer & Awh, 2022; Oberauer & Greve, 2021). The latter point in particular is directly in line with the findings presented here: the effect of proactive interference in immediate memory tasks – that require the binding of words to other words or the association of colors to images – emerges specifically at higher set sizes (Bartsch & Oberauer, 2023).

However, the assumption that WM is immune to PI has also faced opposition from some findings (Beaudry et al., 2014; Ralph et al., 2011). Specifically, Beaudry and colleagues argue that in item-recognition tasks, lures that match a recent earlier trial are harder to reject than new lures. They explain this with a familiarity signal from episodic memory, that lingers across trials and intrudes into the recognition decision. We argue that, when WM is tested with a reconstruction method, as in Experiment 3, or with a continuous reproduction method, as in Experiment 4, where familiarity signals are less helpful, this influence from eLTM plays no noticeable role.

### When does Episodic LTM Contribute to Tests of WM?

Our findings raise the question why episodic LTM traces of pre-learnt information do not contribute to performance at lower set sizes. This is particularly relevant as, in our case, participants could simply draw on the pre-learnt information once they recognized a word pair or image-color pair in the given trial – without the need for encoding a new representation into WM. Instead, the WM system does not access these traces, even though it could positively contribute to performance even at set size 2 (especially in Exp 4). One reason for this might be that the traces in episodic LTM from these stimuli were weaker, or more imprecise, than the traces that could be formed at lower load in WM, preventing them from contributing substantially to performance in an immediate memory test. This is in line with a previously proposed functionality of WM, namely that the exchange of information between WM and LTM is controlled by a flexible gate (Bartsch & Shepherdson, 2022; Oberauer et al., 2017; Oberauer & Bialkova, 2009). Specifically, this hypothesis states that one core function of WM is to form and store representations that deviate from a person’s long-term knowledge (e.g., remembering a friend’s new phone number in order to call them back). Therefore, these representations need to be shielded against interference from LTM and the gate from LTM into WM is controlled such that, by default, the representations in WM guide thought and action. However, in situations where a monitoring process determines that utilizing LTM representations would lead to a better outcome, the gate opens, enabling the WM system to access and utilize information from LTM to guide behavior.

Based on this flexible gate hypothesis, as set size in a WM task increases, there comes a point at which there is more (precise) information available in episodic memory than in WM.^5^ An optimal gate-keeping policy would be to open the gate when that point is surpassed (Oberauer & Bartsch, 2023). That means that in the present study, only when WM representations were inaccessible or below the quality of the information pre-learnt at the beginning of the experiment, the gate to LTM opened, leading to both interference and facilitation effects at set size 4. Instead, at set size 2, the quality of representations in WM was good enough so that the gate remained closed, protecting from proactive interference – but as a downside also from any facilitation effects. This would also explain the ambiguous results with regards to influences of episodic LTM at set size 3 in Experiments 3 and 4: For certain individuals, set size 3 potentially surpassed their WM capacity, resulting in the emergence of proactive interference and proactive facilitation effects specific to them, while these effects were absent in individuals whose capacity accommodated set size 3. Alternatively, for the majority of individuals, set size 3 trials entailed situations where they occasionally possessed sufficiently precise representations for certain trials but not others, thereby leading to intermittent opening and closure of the gate at set size 3.

### Facilitation and Interference effects

Although we were able to generally replicate the finding that information exchange between LTM and WM is limited to situations in which WM capacity is reached, the specific pattern of interference and facilitation of WM performance differed between Experiments 3 and 4. Specifically, Experiment 3 found strong interference effects and smaller facilitation, whereas Experiment 4 found stronger facilitation and smaller interference effects. What could be the reason for this?

In Experiment 3, participants had to remember pairings of words and were immediately tested in a 4-AFC reconstruction task. Prior research has shown that in this type of task, episodic memory already contributes substantially to performance at set sizes larger than 3 (Bartsch & Oberauer, 2023). Specifically, Bartsch and Oberauer argue that each successive pair results in the activation of representations of its elements in semantic memory. In addition to a representation of the current binding within a trial that is encoded in WM, an episodic memory trace is created that represents the elements and their relation as an integrated event (see Cowan, 2019; Cowan & Chen, 2008). This means, that items are represented simultaneously in both eLTM and WM. Based on this assumption, there might have been less room for more facilitation benefits in this task from the pre-learnt representations in eLTM, additionally to the general eLTM contributions. This is further supported by the substantial PI effects observed in Experiment 3, indicating that the gate is typically open due to contributions from eLTM. Consequently, any mismatch between the current information and the stored knowledge in eLTM increases the detrimental effects.

In the visual task of Experiment 4, the colours of the objects are part of a space that has less distinct representations in semantic memory than do individual words. There is evidence that both colours and words have representations that consist of both continuous and categorical components (e.g., Hardman et al., 2017; Trott & Bergen, 2023). However, whereas the number of unique categorical word representations is in the thousands, for colours the number appears to be vastly smaller (e.g., no participant displayed more than 16 unique colour categories in Hardman et al.’s data). This may indicate that continuous aspects of colour representations play a more important role in remembering than is the case for words, but also that different colours are more likely to be attracted to a shared categorical node in semantic memory (e.g., two shades of blue may share a categorical representation; whereas the words “azure” and “blue” have unique, categorical lexical representations). If LTM largely relies on categorical components, the general contribution from episodic and semantic memory in this task used in Experiment 4 might be smaller and less beneficial, meaning the gate to LTM is more often closed than opened. In case a pre-learnt pair is then recognized, subjects can flexibly open the gate and benefit from its facilitative effect. Yet, a mismatch to an item might elicit such an opening less often, thereby reducing its harmful effects.

### What Are Implications for Current Models of Memory?

Our results speak in favor of models that conceptualize WM as a subset of LTM representations (Cowan, 1995; McElree, 1998; Oberauer, 2002), and which propose a flexible interaction of the two systems. In line with these theoretical ideas, we have shown that people can draw on prior knowledge from LTM in tasks of WM in order to extend and overcome their limited capacity. Yet, we have further shown that functionality is limited to situations in which this extension would generally be beneficial, such as when the systems are already interacting because the capacity of WM is exceeded. Such interaction between WM and LTM can yield both benefits, in the form of proactive facilitation, and costs, in the form of proactive interference (PI). The latter occurs when the information currently held in WM diverges from the prior knowledge stored in LTM.

## Conclusion

Across four experiments, we tested three hypotheses pertaining to the conditions under which prior knowledge from LTM contributes to WM. We showed that there are indeed conditions in which only WM contributes to performance: Performance deteriorated with the addition of stimuli from eLTM when WM load was low, but not when it was high; On this basis, it appears that an exchange of information between LTM and WM occurs only when WM capacity is exceeded, with PI and PF effects affecting immediate memory performance in a verbal and visual task only at higher set sizes.

## Data Accessibility Statement

The data and the analysis scripts of all Experiments can be accessed on the Open Science Framework (https://osf.io/spkhb).

## Footnotes

We refer to a “cost of adding LTM information” when adding LTM pairs to a set containing e.g. 2 new pairs (2 + 2 condition) leads to worse memory for those new pairs than when they were presented alone (2 + 0 condition). Importantly, here performance is still higher than for the same number of only new information (4 + 0 condition).

We thank Eren Günseli for pointing us to this explanation.

A model varying kappa over pair types indicated evidence against variations of kappa over pair types and thus we removed this effect from the final model.

As expected, the probabilities for LTM intrusions for simulated LTM colours in the new-new and old-old pair type conditions were virtually zero (for new-new pairs P_LTM_ < 2.3%, for old-old pairs P_LTM_ < 1.4%).

We understand performance in a given test situation is determined by some combination of quality and availability of a memory representation.
